# Olfactory processing in the lateral horn of *Drosophila*

**DOI:** 10.1007/s00441-020-03392-6

**Published:** 2021-01-21

**Authors:** Sudeshna Das Chakraborty, Silke Sachse

**Affiliations:** Department of Evolutionary Neuroethology, Max Planck Institute for Chemical Ecology, Hans-Knoell-Str. 8, 07745 Jena, Germany

**Keywords:** Olfactory coding, Neural circuits, Insect, Hedonic valence, Odor-guided behavior

## Abstract

Sensing olfactory signals in the environment represents a crucial and significant task of sensory systems in almost all organisms to facilitate survival and reproduction. Notably, the olfactory system of diverse animal phyla shares astonishingly many fundamental principles with regard to anatomical and functional properties. Binding of odor ligands by chemosensory receptors present in the olfactory peripheral organs leads to a neuronal activity that is conveyed to first and higher-order brain centers leading to a subsequent odor-guided behavioral decision. One of the key centers for integrating and processing innate olfactory behavior is the lateral horn (LH) of the protocerebrum in insects. In recent years the LH of *Drosophila* has garnered increasing attention and many studies have been dedicated to elucidate its circuitry. In this review we will summarize the recent advances in mapping and characterizing LH-specific cell types, their functional properties with respect to odor tuning, their neurotransmitter profiles, their connectivity to pre-synaptic and post-synaptic partner neurons as well as their impact for olfactory behavior as known so far.

## Introduction

Animals are constantly exposed to an infinite number of complex odor blends in their natural habitat. The identity and specific composition of these odors convey crucial information about food availability, threats from predators or pathogens, mates, and sites for oviposition. Therefore, based on their information content, odors initiate and drive appropriate behavioral responses in animals of highly divergent species. Chemically, these odorants are small, volatile molecules that bind to their cognate olfactory receptors (ORs) expressed in the dendrites of olfactory sensory neurons (OSNs) in the peripheral olfactory organs (Grabe and Sachse 2018). The OSNs, in turn, direct the information to higher-order processing centers in the brain. During this multilayered neural processing, abstract chemical features of odorants are decoded into meaningful neuronal activities. In order to achieve this, the brain transforms the complex sensory input into a neuronal representations of various stimulus parameters, such as e.g., odor identity, odor concentration, and/or hedonic valence (pleasant vs. unpleasant) (Haddad et al. 2008; Knaden et al. 2012; Schmuker et al. 2007). This neuronal mechanism allows animals to detect and discriminate between broad spectra of volatile chemicals in the environment and to accomplish odor-guided decisions. Hence, animals have dedicated brain regions that coordinate the processing of such olfactory signals regarding foraging and feeding, courting with mates, avoiding spoiled or poisonous food, and escaping from predators (Knaden and Hansson 2014). These behavioral choices are often stereotypic and defined as innate odor-driven behavior, in many cases elicited by a highly specific odor stimulus. These innate behaviors are supported by genetically hardwired neural circuits and are crucial for the animals’ survival and reproduction.

Many sensory stimuli exhibit innate valences which can of course differ from animal to animal, such as, e.g., carbon dioxide, which is highly attractive for mosquitoes, but does induce strong repulsion in vinegar flies (McMeniman et al. 2014; Suh et al. 2004; van Loon et al. 2015). However, to behave adaptively in an ever-changing environment, animals must also learn to assign new meanings for sensory stimuli and must “overwrite,” e.g., the valence of an innately attractive and pleasant odor after having a bad experience with it. Indeed, odor-based behavioral decisions have been shown to be modulated by previous experience and associative learning (Davis 2004; Wilson and Stevenson 2003). Vinegar flies for example can easily learn to associate a punishment or a reward with a certain odor and adapt their subsequent behavior accordingly (Fiala 2007). The current assumption is that innate and learned odor representations are coded by divergent neural circuits and processed in distinct brain areas. In insects, the output of the first olfactory center, the antennal lobe (AL; analogous to vertebrate olfactory bulb) is conveyed via different neural pathways—a “memory” and an “innate” pathway—to the mushroom body (MB) as well as the lateral horn (LH), which would be comparable brain structures to the piriform cortex and the cortical amygdala in mammals (Sosulski et al. 2011). The MB is considered to be the key structure for associative learning, memory storage and retrieval (Dubnau and Tully 2001; Heisenberg 2003) and exhibits less deterministic and rather random projection patterns of olfactory inputs from the AL (Caron et al. 2013; Eichler et al. 2017). However, a recent EM connectome dataset of the MB reveals that some PN axon terminals project within the calyx in a stereotyped manner (Li et al. 2020). The LH receives highly stereotypic axonal projections from AL output neurons (Jefferis et al. 2007; Marin et al. 2002; Wong et al. 2002). In recent years, the LH has emerged as the primary signal processing center for coordinating naïve, yet crucial, behavioral responses. However, recent studies have questioned the stringent separation between a “memory” versus an “innate” processing pathway by revealing that context-dependent memory is mediated by neurons in the LH and might be independent of the MB (Zhao et al. 2019). Our knowledge about the role of individual lateral horn neurons (LHNs) with regard to odor coding properties, processing, behavioral impact, and learning has been incomplete due to the unavailability of cell type–specific neurogenetic tools. However, several recent studies in *Drosophila melanogaster* have elucidated to a great extent the anatomy, connectivity, and physiology of diverse LHN populations and therefore extended our current understanding of the processing mechanisms taking place in the LH, which will be summarized and highlighted in this review.

The fruit fly, *Drosophila melanogaster*, is extensively used to investigate the neural mechanisms underlying odor coding and processing due to its anatomically similar, yet simple, olfactory system compared with that of vertebrates (Masse et al. 2009; Wilson and Mainen 2006). The odorant information needs to travel only one synapse to reach from the sensory periphery to the central brain (Su et al. 2009). Moreover, the biggest advantage of this model organism is the ability for selective genetic dissection of the pathways involved. This review mainly focuses on our current understanding of the olfactory circuitry and signal processing mechanisms in the LH, which has recently gained lots of attention as mentioned above. Our aim is to shed some light on how the odor input is conveyed and processed through the LH circuitry to be eventually translated into an appropriate behavioral output. In this review we will begin with a short overview on the transformation of the olfactory code at different neuronal processing layers starting from the peripheral sensory neurons and follow the information flow to higher brain centers. We will describe in detail various aspects of olfactory processing by third-order neurons in the LH, such as the neuronal circuitry, their odor tuning properties, neurotransmitter identity, how these LHNs categorize odor features and finally, their role regarding odor-guided behavior.

## Brief overview of odor transformation from the periphery to higher brain centers

Odor information is encoded at distinct and successive levels of processing that comprise the physico-chemical space, the neural spaces, and finally the perceptual space (Grabe and Sachse 2018; Masse et al. 2009). Various studies have investigated how a chemical signal is translated into a behavioral response while traversing from the sensory input level to higher brain centers using *Drosophila melanogaster* as a model organism.

**Fig. 1:**
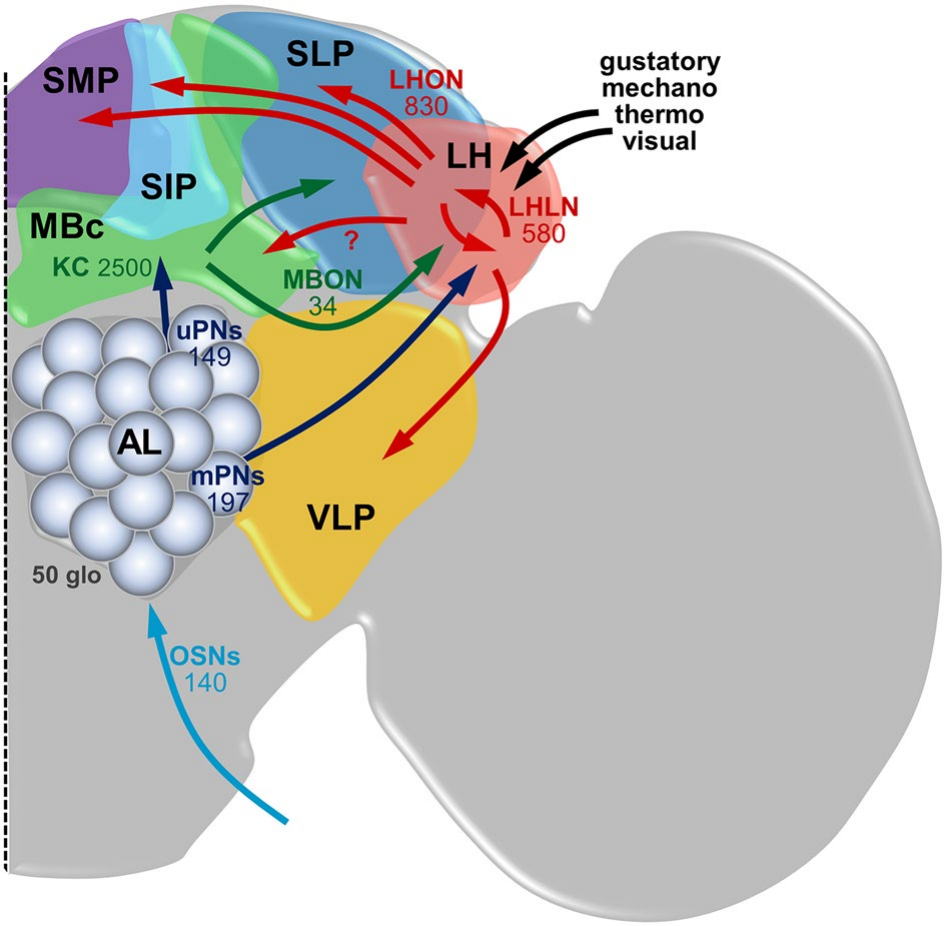
Neuronal circuitry of the major olfactory neuropils in the *Drosophila* brain. Olfactory sensory neurons (OSN) provide the olfactory input to the first olfactory processing center, the antennal lobe (AL) which consists of ~ 50 olfactory glomeruli. From the AL, the olfactory information is conveyed via different populations of projection neurons (uniglomerular uPNs and multiglomerular mPNs) to two second-order brain regions, the mushroom body calyx (MBc), and the lateral horn (LH). The MB is composed of intrinsic neurons, called Kenyon cells (KC), which receive direct PN input. The output of the MB to further brain areas is transmitted by a rather small number MB output neurons (MBONs), of which a few also target the LH. The LH is comprised of local neurons (LHLN) and output neurons (LHON) which relay the olfactory information primarily to the SLP (superior lateral protocerebrum), representing the third-order olfactory centers, as well as to the SIP, SMP (superior intermediate/medial protocerebrum), and VLP (ventrolateral protocerebrum). It is conceivable, but has not been proven yet, that the LH sends feedback information to the MB (indicated by the arrow with question mark). In addition, the LH also receives and integrates input from other sensory modalities. LHONs and MBONs have further interactions in third-order neuropils.

Olfactory processing starts when ORs, expressed within OSNs in the antennae and the maxillary palps, bind to an odorant molecule and transduce this interaction into a neuronal activity, namely, action potentials (Vosshall and Stocker 2007; Wilson 2013). These action potentials are transmitted to the AL, a brain region consisting of ~ 50 olfactory glomeruli that represent the structural and functional units of this primary olfactory center (Fig. 1) (Grabe et al. 2015; Laissue et al. 1999). As a general rule, sensory neurons expressing the same OR converge onto the same glomerulus providing a 1:1 connectivity of OR type input onto single glomeruli (Couto et al. 2005; Fishilevich and Vosshall 2005; Vosshall et al. 2000). In the AL, olfactory information is reformatted and relayed deeper into the brain by projection neurons (PNs), the output neurons of the AL, which number has recently been updated using EM connectomics data and are twice as high as previously reported (Bates et al. 2020). Notably, the olfactory system has evolved different strategies to process odor information: Several odors that are highly crucial for survival and reproduction are encoded by a functionally segregated, so-called “labeled line” pathway—a term adapted from the mammalian taste system—through narrowly tuned ORs leading to a defined and mostly stereotypic innate behavior. These “labeled lines” comprise, e.g., the detection of carbon dioxide (Suh et al. 2004), the male-produced pheromone 11-cis-vaccenyl acetate (cVA) (Kurtovic et al. 2007), the mating enhancing pheromone methyl laurate (Dweck et al. 2015), volatile amines (Min et al. 2013), the odor of parasitic wasps (Ebrahim et al. 2015), and the microbially produced odor geosmin (Stensmyr et al. 2012). On the other hand, the majority of odors including also ecologically relevant odors, such as food and oviposition cues, activate a broad array of ORs and are processed by a combinatorial code to drive various innate behavioral responses (Hallem and Carlson 2006; Schubert et al. 2014; Stökl et al. 2010). Notably, it could be shown that odors that share the same hedonic valence (i.e., that induce either attraction or aversion) activate similar combinations of glomeruli, meaning that odorant valence is encoded by the odor-specific code in the AL (Knaden et al. 2012). This valence-specific code was shown to be more pronounced at the level of the AL output neurons (i.e., PNs) indicating that it emerges from local computations within the AL network. Furthermore, other studies demonstrated that activation of individual glomeruli is already sufficient to mediate innate olfactory attraction or aversion (Bell and Wilson 2016; Mohamed et al. 2019b; Semmelhack and Wang 2009; Suh et al. 2007), while it has also been suggested that a weighted summation of normalized glomerular responses predicts a fly’s innate odor response behavior (Badel et al. 2016; Parnas et al. 2013).

How are the odor-evoked responses of PNs transformed at the next processing level after the pre-processing in the AL? A previous computational modeling study suggested that the odor-evoked AL output patterns would result in highly selective, sparse, and concentration-invariant responses in neurons of the LH (Luo et al. 2010). However, functional studies carried out later on revealed that LHNs exhibit diverse types of response patterns including both narrow as well as broad odor-evoked activities (Fisek and Wilson 2014; Frechter et al. 2019; Kohl et al. 2013; Mohamed et al. 2019a; Ruta et al. 2010). The broadly tuned LHNs receive excitatory input from multiple glomeruli (Bates et al. 2020; Fisek and Wilson 2014). Interestingly, an increase in tuning breadth has also been observed for the PN-LHN transition similar to the OSN-PN synaptic transformation (Frechter et al. 2019). Although the extent of broadening cannot be directly compared between the AL and the LH, it has been suggested that due to direct pooling of feed-forward PN inputs innervating different glomeruli, the broadening might be larger at the LH level (Bates et al. 2020; Frechter et al. 2019). However, also a few narrowly tuned LHNs have been reported that receive excitation from solely a single glomerulus which is gated by strong, odor-selective inhibition from co-activated glomeruli through inhibitory PNs and GABAergic neurons in the LH itself (Fisek and Wilson 2014). In summary, similar to the AL processing mechanisms, odor-evoked responses of PNs are also transformed in a non-homogenous manner at the level of LHNs.

## Neuronal circuitry to the LH

Previous and very recent studies have unraveled the connectivity from the AL to the LH and within the LH. First, we will begin with an overview of the PN-LHN connectivity. Several studies have made predictions regarding which PN-glomeruli combinations might converge onto which LHNs on the basis of their anatomy and target regions in the LH (Jefferis et al. 2007; Silbering et al. 2011; Strutz et al. 2014). PNs from individual glomeruli were shown to project to specific and stereotyped sub-regions of the LH (Jefferis et al. 2007; Marin et al. 2002; Wong et al. 2002) suggesting that odors should be represented in the LH in a similar fashion as reflected by the innervation patterns of the AL. Interestingly though, different studies reached different kinds of conclusions: Studies that dissected the pheromone circuit in *Drosophila* suggested that a certain cluster of LHNs receives input only from a single glomerulus (i.e., in that case the cVA-responding glomerulus DA1) and therefore raised the possibility that each LHN might be highly sparsely and narrowly tuned in its response profile (Kohl et al. 2013; Ruta et al. 2010). At the other extreme, a study in locusts demonstrated that LHNs are broadly tuned and individual LH neurons receive input from several glomeruli (Gupta and Stopfer 2012). A theoretical study pointed towards another alternative, proposing that LHNs perform a complex computation by adding and subtracting sparse, weighted inputs from co-activated glomeruli, which generates highly selective responses to specific odors (Luo et al. 2010). Indeed, functional studies using paired recordings from PNs and LHNs have provided evidence that the above stated possibilities co-exist in the *Drosophila* LH (Fisek and Wilson 2014; Jeanne et al. 2018). These studies revealed further that LHNs receive input from sparse and stereotyped combinations of glomeruli that are co-activated by odors and that certain combinations of glomeruli seem to be over-represented.

Regarding the PN-LHN transformation, Jeanne et al. (2018) mapped the connectivity from individual glomeruli to specific types of LHNs by optogenetically stimulating PNs innervating a single glomerulus while they simultaneously performed whole-cell patch clamp recordings from individual LHNs. This study predicts that the average LHN receives excitatory input from ~ 6.2 glomeruli. This estimation is in line with recent EM connectomics data revealing that LHNs sample the input from on average ~ 6–7 PNs (Bates et al. 2020) and resembles the connectivity of the MB, where each Kenyon cell—the intrinsic neurons of the MB—receives input from an average of 6 PNs (Li et al. 2020). Interestingly, Jeanne et al. (2018) also found that sister PNs (i.e., PNs innervating the same glomerulus) converge onto the same LHN. Such convergence represents a unique feature of the LH and could so far not be observed in the MB of the fly (Caron et al. 2013; Gruntman and Turner 2013). Using NBLAST, an algorithm that allows for measuring neuronal similarities (Costa et al. 2016), a total of 110 LHNs were classified and segregated into 39 morphological types (Jeanne et al. 2018). As expected, LHNs with similar morphologies exhibited similar odor tuning properties and overlapping connectivity. A detailed comparison between the connectivity maps with published OSN and PN odor response profiles (Badel et al. 2016; Hallem and Carlson 2006) resulted in the three following observations: (1) LHNs of the same type receive input from glomeruli with similar odor tuning curves, (2) different LHN types receive input from similar combinations of glomeruli, and (3) glomeruli exhibiting dissimilar odor tuning profiles provide input to similar LHNs (Jeanne et al. 2018). Although all conceivable PN-LHN combinations could be observed in the fly’s LH, a weak correlation and therefore a bias for glomeruli (i.e., PNs) with a similar response profile to target similar LHN types was shown. Such a convergent innervation of glomeruli that are activated by the same odor ligands might represent a coding strategy to improve the signal-to-noise ratio of LHN responses and to increase sensitivity at that processing level.

Notably, glomeruli which are narrowly tuned to odors were over-represented at the level of LHN responses due to their high amount of synaptic connections (Jeanne et al. 2018). In addition, these glomeruli often converged with the output from another glomerulus, forming a glomerular pair that shared a similar meaning. More precisely, these glomerular pairs define behaviorally relevant “odor scenes” which often combine chemically dissimilar volatiles but are linked to similar behaviors, such as courtship, aggregation, or food seeking. Hence, processing of odors with similar behavioral impacts seems to converge on overlapping sets of LHNs. Recently, Huoviala et al. (2018) characterized the “labeled line” processing pathway that is dedicated to the detection of geosmin, an innately aversive odor, starting from the peripheral sensory neurons through PNs and LHNs all the way up to the fourth-order neuron level (Huoviala et al. 2018). Similar to Jeanne et al. (2018), the authors observed a significant divergence of the pathway at the level of PN to LHN connectivity, as well as a convergence of multiple aversive pathways onto the same LHN targets. According to the authors, the divergence of geosmin-responsive PNs to huge and various populations of LHNs seem to define the end of a “labeled line” pathway. That finding raises the question whether the term “labeled line” is still appropriate for OR-glomeruli combinations that are highly specialized in detecting a single odorant ligand, but whose pathway gets broadened at the higher circuit level. However, also neural pathways that are dedicated to a single sensory cue need to be integrated with other sensory modalities and have to be evaluated with regard to the internal state of the animal and its previous experience. For example, the perception of the male-derived sex pheromone cVA in female flies underlies modulation by mating as well as feeding (Das et al. 2017; Lebreton et al. 2014, 2015). In addition, all neuronal circuits eventually need to target and trigger the same or overlapping sets of motor neurons in order to execute a certain behavioral output. It therefore sounds plausible that a “labeled line” code only exists at the level of sensory and second-order neurons where the detection threshold needs to be maximized, while these dedicated pathways feed into a broader network in higher-order brain areas.

## Neuronal circuitry within the LH and beyond

In the next part, we will give an overview of the third-order neuronal circuitry within the LH. The LH is a complex neuropil consisting of ~ 1400 neurons and more than 165 cell types with diverse morphologies (Frechter et al. 2019). In recent years, the functional and anatomical dissection of the LH has been lagging behind compared to other neuropils in the *Drosophila* brain, such as the MBs, due to the lack of neuron-specific and sparse transgenic LH lines. A previous neuroanatomical screen of more than 4000 enhancer trap lines identified only very few neurons with a clear LH innervation (Tanaka et al. 2004). However, very recently, by employing the combination of enhancer-driven expression (Pfeiffer et al. 2008) and the intersectional split-GAL4 system (Dionne et al. 2018; Pfeiffer et al. 2010), Dolan et al. (2019) were able to generate 2444 split-GAL4 lines for LHNs which are sparse yet strong and specifically target 82 different cell types in the LH. Development of these reagents has provided a great opportunity to study identified LHNs for its function mediating various kinds of innate behaviors. Using these driver lines, Dolan et al. characterized three different categories of LHNs: LHONs (lateral horn output neurons), LHLNs (lateral horn local neurons), and LHINs (lateral horn input neurons), which represent mainly PNs conveying the olfactory information from the AL. In total, the LH consists of 580 LHLNs (40%), most of which are inhibitory, and 830 LHONs (60%) (Fig. 1) (Frechter, et al. 2019). Therefore, compared with the AL, the LH has more neurons, in both number and type, than previously expected.

The majority of LHONs transmit the output signal to the next principal node of higher olfactory processing centers, which is the superior lateral protocerebrum (SLP) representing the third-order olfactory processing center. In addition to the SLP, two other nearby neuropils, called the superior intermediate protocerebrum (SIP) and the superior medial protocerebrum (SMP), were also target regions of LHONs (Dolan et al. 2019; Frechter et al. 2019). Olfactory input from the LH and olfactory information from the MB might be integrated in these “convergence zones” (Aso et al. 2014; Dolan et al. 2019). In terms of downstream convergence of LH information, two clusters of LHONs with different neurotransmitter identities co-project to the same location in the SLP, which points towards a possible bidirectional modulation of the same target neuron. Importantly, none of the identified LHONs project either to the ellipsoid body or to the ventral nerve cord, suggesting that at least one or two additional layers of processing must exist before the motor output (Dolan et al. 2019).

Three bilateral LHONs have been observed that are connecting both hemispheres of the brain. These neurons have dendrites in one LH and project to both, the ipsilateral and contralateral output zones, providing a possible mechanism to coordinate the input and information flow of both brain hemisphere and to facilitate an accurate behavioral output (Dolan et al. 2019). Indeed, such a mechanism has been demonstrated by another recent study that characterized odor responses in a defined cluster of third-order neurons, so-called vlpr neurons, which relay information from the LH to the ventrolateral protocerebrum (VLP) (Mohamed et al. 2019a). Using 2-photon functional imaging it could be shown that an asymmetric odor input induces a differential activation of this neuron population in the left and right brain hemispheres due to contralateral inhibition. This mechanism might improve efficient detection of odor concentration gradients and therefore facilitates the navigation capabilities for finding and targeting an odor source.

What is known regarding the information flow between the LH and the MB? LH neurons represent a frequent target of converging MB output neurons (MBONs) (Li et al. 2020), while so far only a few MB-LH connections have been studied in detail. One example represents the LH cell types PD2a1/b1 which are LHONs that are postsynaptic to MBONs, such as MBONs-α2sc. This type of MBON has been demonstrated to be required for the retrieval of aversive olfactory memories, while it is not involved in any appetitive associative learning (Séjourné et al. 2011). Since PD2a1/a2 are postsynaptic to MBONs-α2sc, this LHON population receives learned olfactory information from the MB and is therefore involved in aversive memory retrieval (Dolan et al. 2018). Moreover, it could be shown that these LHONs also receive input from the AL through food odor-encoding PNs, suggesting that they have a dual role regarding aversive memory retrieval as well as innate attraction to certain food odors. Another study has revealed that these MBONs-α2sc neurons are also connected to the so-called MBONγ1pedc>αβ neurons and are involved in food odor tracking (Sayin et al. 2019). According to them depending upon the internal state or previous experience, the MB might control the innate behavioral responses elicited by the LH, confirming again the intense functional connection between the MB and the LH.

In addition to the direct information flow from MBONs onto LHONs, two additional and novel types of convergence onto LHNs have been observed in the larval stage of *Drosophila*: (1) few LHNs are synapsing directly onto MBONs and (2) LHONs and MBONs converge onto the same downstream neurons which therefore function as “convergence neurons,” which often represent a feedback neuron (FBN) that gives direct input onto modulatory neurons (Eschbach et al. 2020). Hence, these findings suggest that many synaptic sites seem to exist, either at MBONs, LHNs, or FBNs, where learned and innate olfactory information could be integrated to determine a behavioral output that has been modulated by previous experience.

What do we know so far about the network within the LH? In comparison to the AL, where various kinds of excitatory as well as inhibitory interactions between the three main neuronal populations (i.e., OSN, PNs, and LNs) have been dissected in great detail and have been shown to be crucial for odor mixture processing, gain control, discrimination abilities, and signal boosting (Barth et al. 2014; Das et al. 2017; Mohamed et al. 2019b; Olsen and Wilson 2008; Silbering and Galizia 2007), the neuronal circuitry within the LH and its functional implications still need to be scrutinized. Recent anatomical studies based on EM connectomics data reveal evidence that LHLNs innervate broad regions of the LH and exhibit potential interactions with various types of LHONs (Dolan et al. 2019), while LHLN-to-LHLN connections are rather sparse (Bates et al. 2020). Notably, both LHONs and LHLNs seem to lack a defined polarization suggesting that information can flow in either direction (Dolan et al. 2019; Frechter et al. 2019). Such a neuronal network organization is similar to the AL, where OSNs and PNs both transmit and receive input from various local interneurons (Das et al. 2017; Liu and Wilson 2013; Mohamed et al. 2019b; Olsen and Wilson 2008; Root et al. 2008; Yaksi and Wilson 2010). According to Dolan et al. (2019) and Bates et al. (2020) LHINs relay multisensory inputs from the visual, mechanosensory (Patella and Wilson 2018), gustatory (Kim et al. 2017), and thermosensory (Frank et al. 2015) system to a restricted ventral zone of the LH. Therefore, the LH can be divided into two domains, a multimodal ventral zone as well as a dorsal zone which is mainly restricted to olfactory inputs. In addition, Dolan et al. (2019) also identified a putative ascending neuron sending input from the ventral nerve cord to the LH, which might convey mechanosensory or pheromonal information (Ramdya et al. 2015; Thistle et al. 2012). The neuroanatomical groundwork from the EM connectomics data provides a huge amount of detailed information regarding the LH circuitry and an excellent basis for future studies examining the functional network properties and its impact regarding odor-guided behavior.

## Odor tuning by LHNs

Based on anatomical data of individually traced PNs, several previous studies have predicted odor-evoked activation maps in the LH. The stereotyped branching patterns of PNs in specific and distinct regions of the LH have led to the assumption that LHNs exhibit reproducible odor-evoked responses that are conserved from animal to animal (Jefferis et al. 2007; Tanaka et al. 2004) and that these third-order neurons integrate an input across multiple olfactory input channels defined by the odor ligand profile of the corresponding OR. Later on, functional studies have demonstrated clearly that LHNs respond indeed in a reproducible and stereotyped manner to all odors and that this stereotypy is not confined to only pheromone-responsive neurons, as previously assumed, but rather represents a general feature of the LH (Fisek and Wilson 2014; Frechter et al. 2019). Based on the odor tuning properties, LHNs were initially classified into two categories: first, narrowly tuned LHNs with sparse and highly selective odor response profiles (Fisek and Wilson 2014; Kohl et al. 2013; Luo et al. 2010; Ruta et al. 2010) and second, broadly tuned LHNs which are functionally more heterogeneous (Fisek and Wilson 2014; Frechter et al. 2019). However, when Frechter et al. (2019) screened the odor response profile of 242 LHONs and 84 LHLNs, they were able to classify 64 distinct functional cell types based on the specific odor tuning profiles determined with an array of 36 odors. When they took both, the anatomical and the odor tuning into account, they observed that many morphologically distinct LHNs exhibited a similar odor response profile. This is in contrast to the findings from Jeanne et al. (2018), where they reported that morphologically similar LHNs exhibit rather similar tuning patterns.

In general, LHON’s spontaneous firing is ten times lower compared with the firing rate of PNs, while they respond to, on average, three times more odors than PNs, but with a lower firing rate (Frechter et al. 2019). These findings indicate that LHNs exhibit generally broader tuning patterns than their presynaptic partner neurons and are therefore less odor-specific. Although excitatory PN-LHN connections are the major driver of LHN odor responses, some specific properties of the odor-evoked LHN responses must arise from other additional neuronal connections (Jeanne et al. 2018; Liang et al. 2013). One possible explanation for the different response properties of the presynaptic and postsynaptic partner neurons (i.e., PNs and LHNs) could be that LHNs perform analogous computations by integrating distinct glomerular inputs in a supralinear manner (Fisek and Wilson 2014; Jeanne et al. 2018; Jeanne and Wilson 2015), meaning that certain combinations of odors evoke synergistic rather than linear mixture responses as also shown for some odor mixtures in the AL (Das et al. 2017). Hence, the response of some specific LHN types would consequentially be more robust than their constituent neurons to certain odor ligands and therefore elicit a stronger behavioral output response (Jeanne et al. 2018). This scenario arises due to the biased convergence of some specific pairs of glomeruli sharing the same “odor scene” feature that are linked to certain behaviors as mentioned before. Such a biased connectivity pattern provides the mechanistic basis for coding certain odor features rather than odor identity and therefore facilitates odor categorization. However, there is still some debate going on about which odor features are being represented and encoded in the LH. Several theories have emerged over recent years based on various experimental approaches and observations: First, tracing of the axonal target regions of excitatory PNs in the LH revealed that PNs responding to pheromones innervate a distinct LH part, while PNs detecting food odors innervate another (Jefferis et al. 2007). Hence, this early study suggested already that odors are organized according to behavioral significance in the LH. Second, another study based on silencing MB function concluded that the LH might mediate innate responses to repulsive odors only (Wang et al. 2003), which could not be confirmed by functional studies examining odor representations in the LH that were carried out later on (Parnas et al. 2013; Strutz et al. 2014). Third, functional imaging studies linked to innate olfactory behavior revealed that the LH is encoding hedonic valences and odor intensity as a spatially segregated functional map (Strutz et al. 2014) and therefore provided the first functional evidence for an activation map based on odor categorization in the LH. These observations are largely in line with two recent studies showing that odor representations in the LH are based on complex behavioral “odor scenes” (Jeanne et al. 2018) or organized according to the chemical groups of odor ligands (Frechter et al. 2019). Hence, all these studies mentioned agree on the concept that the LH is categorizing odors according to behavioral values, which stands in strong contrast to the combinatorial odor-specific glomerular activity map of the AL (Grabe and Sachse 2018).

It is important to mention that the categorization of odor features in the LH in form of hedonic valence seems to be plastic and modulated by the internal state of the animal. A specific cluster of LHONs, the above-mentioned PD2a1/b1, have been shown to assign valence in a context-dependent manner. PD2a1/b1 LH neurons were demonstrated to promote approach behavior at low odor concentrations in starved flies (Dolan et al. 2018), while they contribute to avoidance behavior at high odor concentrations in satiated flies (Lerner et al. 2020). However, it still remains elusive how the LH circuitry facilitates odor categorization to decode useful information in the form of behavioral value.

## Neurotransmitter identity and their impact

**Fig. 2:**
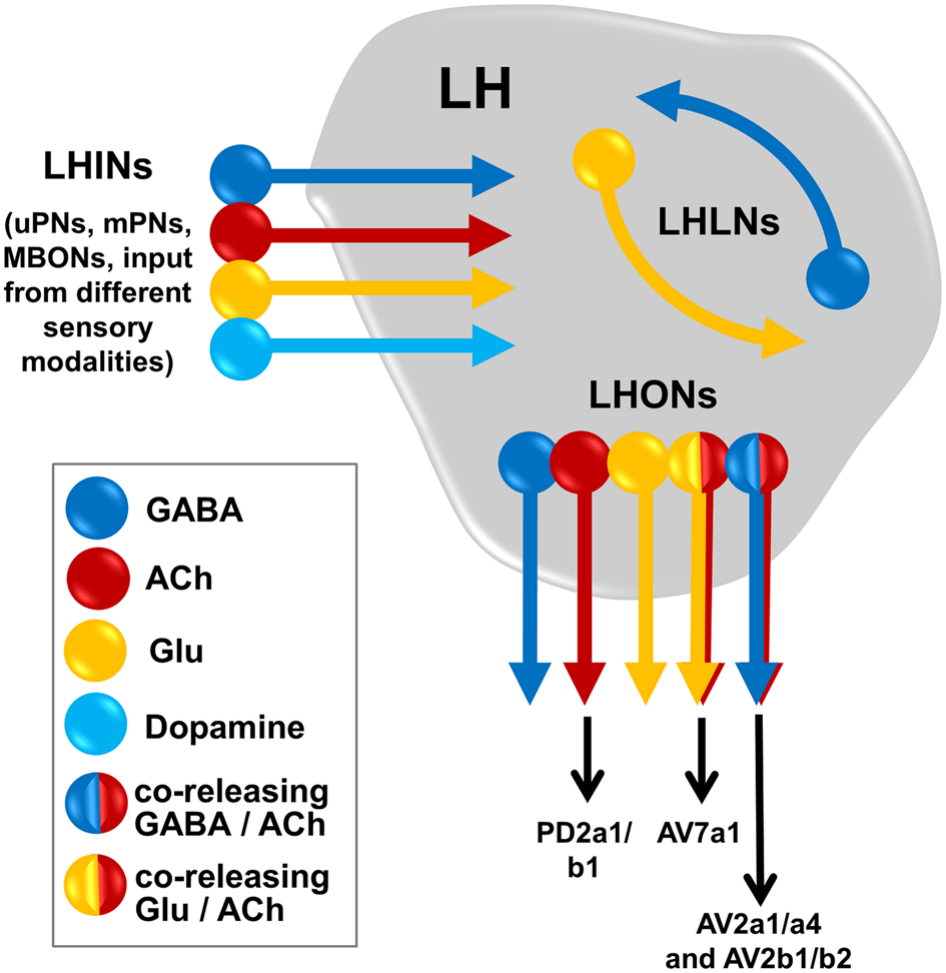
Schematic depiction of the neurotransmitter profile of the various neurons innervating the LH. Lateral horn local neurons (LHLNs) and lateral horn output neurons (LHONs) receive olfactory input from lateral horn input neurons (LHINs) which are mainly comprised of olfactory PNs (uniglomerular and multiglomerular), MBONs, and neurons from other sensory modalities. Different colors indicate the neurotransmitter identity of the neurons shown. The so-called PD2a1/b1 neurons, which represent LHONs, have been shown to be cholinergic. Few LHONs, such as AV2a1/a4, AV2b1/b2, and AV7a1 co-release two neurotransmitters

The existence of ~ 1400 neurons in the LH in addition to their diverse neurotransmitter profiles makes the LH circuitry even more complicated and impedes dissecting the role of individual neuronal LHN populations regarding odor processing and odor-guided behavior. What do we know so far regarding the neurotransmitter profiles of the various LHN types? A previous study characterized one population of GABAergic local neurons in the LH and classified LHNs into two categories, one broadly and one narrowly tunes LHN type, as mentioned above (Fisek and Wilson 2014). Recently, the study by Dolan et al. (2019) has shown the existence of cholinergic, GABAergic, and glutamatergic populations of LHNs. According to their study, LHONs, LHINs, and LHLNs can be clustered into groups based on their neurotransmitter identity (Fig. 2). Several distinct populations of cells, confined only to the LH (i.e., LHLNs), release GABA or glutamate, while LHONs could be identified for all three neurotransmitters analyzed. Dolan et al. (2019) characterized the neurotransmitter profile of 44 LHONs and 8 LHLNs, of which 26 LHONs were cholinergic, 13 LHONs/3 LHLNs were GABAergic, and 9 LHONs/5 LHLNs were glutamatergic. This finding denotes that the target neuropils of LHONs should receive both kinds of input, i.e., excitatory as well as inhibitory. Notably, three cell types were shown to have a dual neurotransmitter profile, whereby two cell types (AV2a1/a4 and AV2b1/b2) were both cholinergic and GABAergic, while the so-called AV7a1 was cholinergic and glutamatergic indicating that these LHONs give inhibitory as well as excitatory output to postsynaptic partner neurons (Dolan et al. 2019). It is assumed that these neurons might have potential interactions with many LHON dendrites. These data indicate that, in addition to the lateral inhibition shown by Fisek and Wilson (2014), lateral excitation via excitatory glutamatergic signaling (Miyashita et al. 2012) might also occur in the LH. As a side note, it should be mentioned that glutamate can have various kinds of impact (excitatory, inhibitory or as coincident detector) depending upon the postsynaptic receptors on their downstream target neurons (Das et al. 2011; Liu and Wilson 2013; Miyashita et al. 2012). Hence, the presence of this huge amount of glutamatergic neurons in the LH suggests a far more complicated neuronal signaling mechanism than simple excitation in the LH. Furthermore, it has been shown recently that the axon terminals of dopaminergic neurons belonging to the so-called PPL2ab cluster also target the LH (in addition to the MB calyx), which indicates that complex neuromodulation complements excitatory and inhibitory interactions at the LH level (Li et al. 2020). However, it is so far unknown whether other neurotransmitters (such as dopamine or octopamine) or neuropeptides are also released by LHNs themselves.

Another neuron of interest that projects also to the LH represents the serotonergic deutocerebral neuron (defined as CSDn) which connects the first and second olfactory centers in the fly brain (Coates et al. 2020). A recent study showed that the CSDn exhibits distinct and opposing odor-evoked responses within the different neuropils innervated, i.e., the AL and the LH (Zhang et al. 2019). The CSDn processes in the AL are generally inhibited by odors, whereas they are excited in an odor-specific manner in the LH indicating that neuronal branches belonging to the same neuron can act differently depending on the brain area innervated.

It would be important to address in future studies the identity of the postsynaptic partner neurons of these identified LHNs in order to understand how these LHNs modulate the activity of these neurons in the next processing level. Altogether the knowledge of the neurotransmitter profile of various LHNs opens up the opportunity to study the complex network in the LH and its underlying neuronal circuit function in order to decode the incoming olfactory information from second-order neurons.

## Role of the LH regarding odor-guided behavior

Since the higher brain regions possess distinct anatomical and physiological properties, it is likely that their neurons might have distinct functions with regard to olfactory behavior. The majority of studies that have employed direct functional manipulations were exclusively limited to the understanding of the function of the MB and were lacking for the circuitry of the LH until recently. Using split-GAL4 lines it could now be demonstrated that the activity of LHNs can be stereotyped depending upon the odor category and different aspects for odor-guided behavior (Frechter et al. 2019). The same odors in different ratios or combinations were shown to elicit different behaviors by targeting different pre-motor circuits. Using artificial activation by targeted expression of an optogenetic effector (such as CsChrimson) (Klapoetke et al. 2014), several LHNs have been identified whose activity can drive innate attraction or aversion and can also lead to changes in motor behavior (Dolan et al. 2019). A specific LHN cell type (so called AV1a1) has been recognized in olfactory behavior for egg-laying aversion (Huoviala et al. 2018) induced by the detection of the toxic mold odorant geosmin (Stensmyr et al. 2012). Similarly, activity in two sets of LHNs (LHON and LHLN) has been demonstrated to be required for the complete behavioral response to carbon dioxide (Varela et al. 2019).

Although earlier studies indirectly implicated a function of the LH regarding solely innate olfactory behavior (de Belle and Heisenberg 1994; Heimbeck et al. 2001), recent emerging evidence has indicated that LHNs play also a crucial role for learned behavior. In support of the role of LHNs regarding learned behavior is the fact that LHONs connect the LH with the SLP and neighboring neuropils; all these brain regions are also targeted by MB-associated neurons, indicating a potential coordination between innate and learned odor responses (Dolan et al. 2018). Recently, a specific class of LHNs (defined as PD2a1/b1) has been reported to perform a dual behavioral role by integrating both innate (from the AL) as well as learned olfactory information (from MBONs) of specific valence; these LHNs are required for innate approach responses at low odor concentration as well as learned aversive retrieval (Dolan et al. 2018). At high odor concentrations, the same neurons contribute to innate avoidance responses in satiated flies, as mentioned above (Lerner et al. 2020). Hence, this circuit provides a mechanism by which learned and innate olfactory information can interact and be integrated in identified neurons (i.e., PD2a1/b1) which then contribute to either attraction or aversion behavior in a context-dependent manner. Another cluster of LHONs, so-called AD1b2, have been found to interact with three types of MBONs to drive approach behavior when activated (Dolan et al. 2019). Another study reported a subgroup of LHNs, which is necessary for recall of protein synthesis-independent, but context-dependent LTM and therefore provides the first evidence that the LH is involved in memory formation (Zhao et al. 2019). Frechter et al. (2019) and Bates et al. (2020) have also identified multiple sites where LHN axon terminals receive input from MBONs, supporting the idea that the MB in general largely interacts and modulates innate olfactory pathways which are critical for learned behavioral recall. These findings emphasize the extensive interconnection between the two higher brain centers, the MB and the LH, whose function with regard to olfactory behavior was apparently underrated in previous studies. Nevertheless, summarizing the results from all recent LH studies supports the hypothesis that stereotyped integration enables an odor categorization at the LH level to evaluate both learned and innate odors whereas the MB may rather encode the identity of the learned odor. However, it still remains elusive which odor representation represents the final readout and where the behavioral output gets determined.

## Conclusion

The LH has emerged as the center for integrating innate behavioral responses and shares many similarities with the mammalian cortical amygdala, since it receives spatially stereotyped input from individual glomeruli of the olfactory bulb and is also involved in processing odor information that directs innate odor-guided behavior (Miyamichi et al. 2011; Sosulski et al. 2011). In recent years, the LH has gained increasing attention and the generation of LH-specific split-GAL4 lines in combination with EM connectomics data of the adult fly brain has strongly advanced our understanding of LH neuron types and their putative function. We know by now that the LH exhibits different coding strategies for odor inputs than the first olfactory center, the AL, as it reveals a categorization of behavioral values by encoding certain odor features instead of odor identities. The LH comprises stereotyped and rather chemotopic circuits, while the MBs represent a rather randomly organized brain area. Although our knowledge about LH neurons has been widely extended in recent years, we still lack the full picture and additional studies are strongly required in the future to understand the complete LH circuitry and its impact regarding odor-guided decisions.
